# Measurement of Hansen Solubility Parameters of Human Stratum Corneum

**DOI:** 10.22037/ijpr.2019.112435.13755

**Published:** 2020

**Authors:** Negin Ezati, Michael Stephen Roberts, Qian Zhang, Hamid Reza Moghimi

**Affiliations:** a *Department of Pharmaceutics and Pharmaceutical Nanotechnology, School of Pharmacy, Shahid Beheshti University of Medical Sciences, Tehran, Iran. *; b *Therapeutics Research Centre, School of Medicine, Translational Research Institute, University of Queensland, Brisbane, QLD, Australia. *; c *School of Pharmacy and Medical Sciences, University of South Australia, Adelaide, SA, Australia.*; d *Protein Technology Research Center, Shahid Beheshti University of Medical Sciences, Tehran, Iran.*

**Keywords:** Human skin, Stratum Corneum, Drug Permeation, Polarity, Hansen Solubility Parameters, Partitioning

## Abstract

Hansen Solubility Parameters (HSP) of human stratum corneum (SC) represent its polarity and are very important for design and optimization of dermatological formulations. However, there is no directly measured data available in the literature for such a crucial property, which is the subject of the present investigation. HSP of the SC was measured by solvent uptake here. 18 solvents/mixtures with different HSP values were selected and their uptake by the SC was measured at 32 °C. The solvents were then divided into good and bad solvents according to their uptake into the SC. The HSP discrete parts of “atomic dispersion forces (δD)”, “dipolar intermolecular forces (δP)”, and “hydrogen bonding (δH)” were then calculated using uptake data and HSPiP software. Results showed that δD, δP, and δH values of the SC at 32 °C are 16.5, 12, 7.7 respectively. The obtained HSP values, which were measured for the first time here, were then used to interpret enhancement effects of permeation enhancers and the uptake of vehicles by the SC using Relative Energy Difference (RED), with good correlations. SC HSP values can be further used in transdermal drug delivery, cosmetic formulations, safety issues, etc.

## Introduction

Human skin can serve as a pathway for noninvasive drug delivery for dermal and transdermal purposes (1). Despite advantages of this pathway, many drugs and particles cannot permeate the skin barrier in therapeutic amounts mainly due to barrier properties of the stratum corneum (SC) (2). The SC contain keratin rich corneocytes (brick) embedded in an intercellular lipid mortar (3) of which the intercellular lipids provide the main pathway for absorption of most drugs (3-5).

The drugs with low affinity (partitioning) to the SC usually show low percutaneous absorption. Partitioning depends on the polarity of the barriers (6) and therefore, for optimized transdermal drug delivery, the polarity of the SC should be understood. One of the best methods for describing the polarity of a barrier is the solubility parameter, which is the subject of the present investigation.

Many methods have been developed for decades to predict solubility parameter. Hildebrand solubility parameter was raised first (7) and then its shortcomings were optimized by Hansen. The Hansen solubility parameters (HSP) are based on the cohesive energy that arise from non-polar or (atomic) dispersion forces (δD), permanent dipole-permanent dipole (dipolar intermolecular) forces (δP), and hydrogen bonding (δH) (8).

HSP can be used for prediction of affinity of material to each other, including drug delivery systems. For example, HSP has been used for drug design for nail disorders (9) and prediction of drug distribution in microspheres (10). The correlation between HSP of two systems (comparison) is evaluated by calculation of Ra (HSP distance) (Equation 1), a modified difference between the HSP of the systems (8). Two systems with close HSP values show smaller Ra and more likely to be compatible; like likes like, like attracts like or like dissolves like.

Ra^2^ = 4(δD_2_ - δD_1_)^2^ + (δP_2_ - δP_1_)^2^ + (δH_2_ - δH_1_)^2^

Equ. 1

HSP could be determined experimentally by observing the interactions between the test material and solvents with defined HSP by, e.g., uptake studies, or other methods (11). Hansen solubility parameters for a lot of materials are available including gases such as carbon dioxide, solids such as carbon-60, sugar, DNA, proteins, and biological membranes (12-14).

In spite of its importance, there is no experimentally measured data available for HSP values of normal human or animals SC. However, there are two HSP values available for human epidermis (8) and psoriatic scales (13). Human epidermis (not the SC) data was evaluated by Hansen (8), using permeation data reported by Ursin *et al*. (14). Another available set of data, that is an estimation, is reported by Abbott (15). The aim of this investigation is to measure HSP values of the human SC directly for the first time.

## Experimental

*Material*

Trypsin was purchased from Sigma-Aldrich. Dimethyl solfoxide and dimethyl ether were obtained from ChemLAB (Belgium). Methanol, ethanol, acetonitrile, n-hexane, tetraheydrofuran, chloroform, dichloromethane, ethyl acetate, 2-propanol, 1-butanol, toluene, l-menthone and N,N-dimethylformamide were obtained from Merck (Germany).

*Stratum corneum preparation*

Human abdominal skin was obtained from cosmetic surgical procedures, following approval by the Shahid Beheshti University of Medical Sciences Ethics Committee. The donors were all females, aged between 35 and 45 years. The subcutaneous fat of skin samples was removed and the skin was stored at -20 °C. For SC isolation, the frozen skin was thawed to room temperature, washed with distilled water and used for SC preparation. The skin sheets were then immersed into 60 °C purified water for 60s to separate the epidermis (16). Subsequently, the epidermis was transferred into a petri dish containing trypsin solution and sodium bicarbonate (pH = 8) and incubated overnight. After this step, the epidermis was washed away and the remaining sheet (the SC) was dried and stocked at room temperature until use.

*Stratum corneum HSP measurements*

Hansen solubility parameters (HSP) of the SC were measured by solvent uptake method. The SC samples were submerged in the selected solvents with different HSP values (Table 1) at 32 °C for 12h and solvent uptake was measured gravimetrically and HSP was calculated, as discussed below.

*Solvent selection*

To obtain HSP, the SC should be exposed to different solvents with higher and lower HSP values in comparison to skin. As there is no data available for human SC, the values of δD = 17.6, δP = 12.5 and δH = 11 (8), that are estimated for human epidermal membrane, were used as a reference point here and the solvent were chosen in a way to have HSP values of close, lower and higher than the reference points (Table 1).

*Uptake studies*

Gravimetric method was used to measure the uptake of solvents by the SC. For this purpose, the SC was weighed and then placed individually in glass vials containing solvent and the vials were then placed at 32 °C for 12 h. The SC samples were then removed, dabbed dray, and weighed. The weight differences after correction were considered as the amount of solvent picked by the SC (Equation 2):

%Solvent uptake = (Final SC weight - Initial SC weight)/(Initial SC weight) × 100

Equ. 2

*HSP calculation*

Based on the uptake by the SC, each solvent was first given a score to discriminate between good and bad solvents. The uptake scores were then input into the HSPiP software (obtained from Hansen-Solubility website) together with δD, δP, δH of all solvents. This software plots a 3-dimensional graph in which a ‘sphere’ that includes the ‘good’ solvents is located (see results for a sample plot). The center coordinates of the sphere give the HSP values (δD, δP and δH) of the test material (the SC in this study).

## Result and Discussion

Table 2 shows the SC solvent uptake at 32 °C, representing values of as high as 600% and as low as 20%, indicating a wide range of solvent uptake and therefore, proper solvent selection. According to the results, solvents such as DMSO and its mixture with diethyl ether showed the highest affinity to the SC.

According to the HSPiP software instructions, each solvent can be assigned with a score between 1 (good solvent) and 6 (bad solvent) or any score in between. In this investigation, solvents such as DMSO and its mixture with uptake values of about 300% and higher were considered as good solvents and were given scores of 1. Solvents with uptake values of less than 100% such as toluene, acetone, and ethyl acetate were considered as bad solvents and were given scores of 6. Other solvents were given scores of 2, 3, 4, or 5 based on their uptake values.

**Figure 1:**
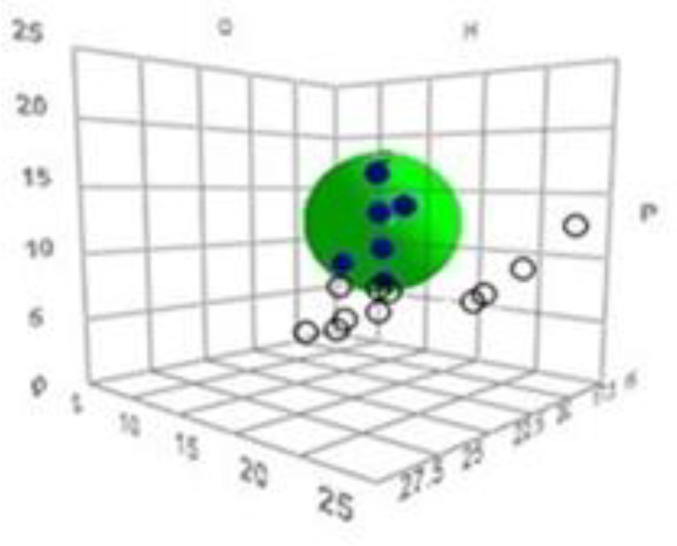
3D solubility bodies of the SC 32 °C and the position of solvents (●: inside of sphere and ○: outside of sphere) obtained from HSPiP. The Center of the spheres shows the HSP values for the SC: δD = 16.5, δP= 12, δH = 7.7

Figure 1 shows the obtained 3D solubility body provided by HSPiP. The data fit of 1.0 (given by the software) indicates that there is perfect separation of good solvents from bad ones by a “spherical” HSP correlation (Figure 1).

Table 3 shows the calculated HSP values for the human SC at 32°C (δD = 16.5, δP = 12, δH = 7.7). Ro (the radius of interaction sphere) was also calculated to be 6.3. There is no data available for HSP values of the human SC in the literature, as discussed in the Introduction. Hansen has measured HSP values of human skin using permeation data through living human skin (14). Our data show partial agreement with Hansen data in terms of δD and δP (see Table 3), but indicate a much lower δH (7.7 *vs* 11). This might be due to the higher H-bonding capacity of skin in comparison to the SC, considering their structure and composition (17).

Two other sets of data are also available, that are HSP values of human psoriatic scales measured through swelling studies (24.6, 11.9 and 12.9 for δD, δP, and δH, respectively) (13) and an estimated system of 17, 8, 8 (for δD, δP and δH respectively) for normal human skin (15). These two sets of data show some differences to our SC data, as expected, because, the natures of systems or measurement methods are different from those of the SC used in the present investigation. As the method employed here (uptake studies) is a proper representative for polarity, our data might be considered as the most relevant HSP values for the human SC, the major barrier to transdermal drug delivery.

*Application of the measured HSP*

The present investigation provides a good tool to discriminate solvents based on their uptake potentials. Such data can be used for optimizations of vehicles (e.g. nanoparticles), enhancers and drugs for required stratum corneum uptake and therefore, action, as explained below.

*Correlation HSP and skin permeation/enhancement*

Enhancement effects of chemical penetration enhancers are mostly related to their effects on and therefore uptake by the SC (18). Accordingly, the increased uptake of the enhancers in the SC is generally expected to increase their action. The same might be applied to vehicle uptake and permeation. Therefore, it was decided here to examine this concept using the HSP values of the SC measured here.

To investigate the correlation between the skin permeation/enhancement and HSP values, we need Relative Energy Difference (RED) (Equation 3), that is the ratio of Ra (see Introduction) and Ro (the radius of interaction sphere). This equation can predict affinity of material to each other. Thus, RED close to or lower than 1 indicates high affinity and RED values of noticeably greater than 1 indicate low affinity (see (8).

RED = Ra/Ro 

Equ 3

The above concept and equation were used here to interpret some skin permeation/enhancement studies, as follows. Lee *et al*. (19) used combination of ethanol and tricaprylin for the skin permeation of tegafur (Table 4). We calculated RED of these systems and a good correlation between flux and reduction of RED was observed (Table 4); the higher the RED value, the lower the flux.

In another study, a combination of isopropyl myristate (IPM):n-methyl pyrrolidone (NMP) (25/75) signiﬁcantly improved lidocaine ﬂux across human skin (25-fold) in comparison to isopropyl myristate alone (20). Using HSP and Ro values obtained in this study, the RED values of IPM/NMP and IPM were calculated to be 0.4 and 1.7 respectively, indicating that the affinity of combination to the SC is higher, in good correlation with permeation data (Table 5).

Also in another study, the combination of IPM with 2-(2-Ethoxyethoxy) ethanol (DEGEE) (40/60) showed increased skin permeation of clebopride (80-fold) compared to IPM alone (21). RED of these combinations was calculated here and showed good correlation with the flux values; lower RED shows higher flux (Table 5).

These above-mentioned data and discussion show that increased affinity of vehicle/enhancers to the stratum corneum increases their permeation/enhancement effect and that a good correlation is observed with HSP values through RED, indicating that HSP is a good tool for prediction of permeation/enhancement effects. However, we should note that vehicles/enhancers act through different mechanisms of which some might be independent from uptake e.g. drug-vehicle/enhancer complexations. Besides this, the enhancement effect of some permeation enhancers does not show direct relationship with their concentration in the system, such as what was reported by Moghimi *et al*. who showed that increased concentration of some terpenes reduces their enhancement effects in model membranes (22, 23). In such cases, enhancement effects are not expected to correlate with RED.

*Vehicle design for better uptake into the SC*

It is well known that passive drug permeation through biological barriers depends on the membrane/vehicle partition coefficients. It can be imagined that beside drugs, the vehicle itself can also partition into the membrane. In such a consideration, HSP values can be used to optimize vehicle uptakes and therefore permeation of their cargos. In this direction, Roberts group studied permeation of caffeine and naproxen using nanoemulsion systems containing oleic acid and eucalyptol and found that both nanoemulsions increased permeation of drugs in comparison to control solutions of water and water/ethanol mixture (24). They also found that highest uptake of formulations into the SC corresponds on the lowest HSP distance (Ra) using HSP values of δD = 17, δP = 8, and δH = 8. RED values of their two nanoemulsion systems and two control solutions were calculated here using Ro and HSP values obtained for human SC in the present investigation. Results showed RED values of 5.5, 3.4, 2.3, and 2.2 for water, water/ethanol (40/60), oleic acid nanoemulsion, and eucalyptol nanoemulsion, respectively. These RED values correlate with uptake of formulations into the SC and the conclusion that they made from Ra values (24). 

**Table 1:** Selected solvents and their HSP values. HSP data are obtained from (8)

**Solvents**	**HSP (MPa½)**		
	**δD**	**δP**	**δH**
DMSO	18.4	16.4	10.2
Diethyl ether/DMSO 25/75	17.4	13.0	8.9
Diethyl ether/DMSO 50/50	16.4	9.6	7.6
Diethyl ether/DMSO 75/25	15.4	6.2	6.3
Dichloromethane	17.0	7.3	7.1
Methanol	14.7	12.3	22.3
2-Propanol	15.8	6.1	16.4
Ethanol	15.8	8.8	19.4
n-Hexane	14.9	0	0
Acetonitrile	15.3	18	6.1
Chloroform	17.8	3.1	5.7
Diethyl ether	14.5	2.9	4.6
Ethyl acetate	15.8	5.3	7.2
Tetrahydrofuran	16.8	5.7	8
N, N-Dimethylformamide	17.4	13.7	11.3
1-Butanol	16.0	5.7	15.8
Toluene	18.0	1.4	2
l-Menthone	17	8.1	4.1

**Table 2:** **Solvent uptake of the SC following 12 h incubation with solvents at 32 °C (data are mean ± SD, n = 5)** **.**

**Solvents**	**Uptake (% w/w)**
DMSO	620 ± 36
Diethyl ether/DMSO 25/75	596 ± 34
Diethyl ether/DMSO 50/50	506 ± 10
Diethyl ether/DMSO 75/25	392 ± 8
Dichloromethane	197 ± 17
l-Menthone	488 ± 12
N, N-Dimethylformamide	473 ± 22
Methanol	170 ± 24
2-Propanol	150 ± 21
1-Butanol	290 ± 11
Ethanol	151 ± 9
n-Hexane	65 ± 6
Acetonitrile	189 ± 7
Chloroform	20 ± 4
Diethyl ether	22 ± 4
Toluene	56 ± 5
Tetrahydrofuran	65 ± 8
Ethyl acetate	52 ± 12

**Table 3:** Calculated HSP values of the stratum corneum (SC) at 32 °C in the present investigation in comparison to HSP values of viable epidermis and psoriatic scales obtained from the literature

**Sample**	**HSPs (MPa½)**			
	**δD**	**δP**	**δH**
SC (present study)	16.5	12	7.7
Human skin ^1^	17.6	12.5	11
Human skin ^2^	17	8	8
Psoriatic scales^ 3^	24.6	11.9	12.9

^1 ^Data are from (8)

^2^ Data are from (15)

^3^ Data are from (13)

**Table 4:** Calculated RED for different ratio of ethanol/tricaprylin and skin permeation parameters of tegafur from ethanol/tricaprylin

**Ethanol/tricaprylin ratio (v/v)** ^1^	**Flux (µg/cm** ^2^ **/h)** ^1^	**RED**
100/0	11.2	2.0
80/20	68.5	1.6
60/40	99.2	1.3
40/60	179.9	1.1
20/80	137.9	1.2

^1^ Data obtained from (19).

**Table 5:** Calculated RED of isopropyl myristate (IPM) and its combination with n-methyl pyrrolidone (NMP) and 2-(2-Ethoxyethoxy) ethanol (DEGEE) in relation to permeation of the penetrants. See text for details

**Vehicle**	**Ratio(v/v)**	**Flux (µg/cm** ^2^ **/h)**	**RED**
IPM	100/0	1.98^1^	1.7
IPM/NMP	25/75	56.7^1^	0.4
IPM	100/0	0.58^2^	1.7
IPM/ DEGEE	60/40	46.35^2^	0.9

^1^Data are for lidocaine ﬂux across human skin from (20).

^2^Data are for skin permeation of clebopride from (21).

## Conclusion

HSP values of the SC, which are measures of its polarity and affinity to drugs, enhancers and vehicles, were measured here for the first time at SC natural temperature (32 °C). These values can be used as a tool to envisage drug/vehicle/enhancer uptakes by the SC. Such data are useful in optimization of drug/formulation for transdermal drug delivery and enhancement and even prevention of percutaneous absorption of hazardous chemicals.

However, we should note that transdermal drug delivery is a complex phenomenon and depends on different variables of which one is defined by affinity. HSP define the system for affinity and might not be able to provide answer to the questions that are related to other properties such as diffusion coefficient.

Further investigations are in progress in our laboratories to explore other aspects of stratum corneum HSP such as effects of temperature, humidity, etc. The same type of investigation should be performed on damaged skin such as burn eschar, as damaged skin behave differently from normal skin in terms of drug partitioning, permeation, and enhancement (25).
